# Implementing community participation via interdisciplinary teams in primary care: An Irish case study in practice

**DOI:** 10.1111/hex.12692

**Published:** 2018-05-16

**Authors:** Edel Tierney, Rachel McEvoy, Ailish Hannigan, Anne E. MacFarlane

**Affiliations:** ^1^ Graduate Entry Medical School University of Limerick Limerick Ireland; ^2^ Health Service Executive Galway Ireland

**Keywords:** community participation, community representatives, health services, interdisciplinary teams, normalization process theory, planning, primary health care

## Abstract

**Background:**

Community participation in primary care is enshrined in international and Irish health policy. However, there is a lack of evidence about how stakeholders work collectively to implement community participation within interdisciplinary teams; community perspectives are rarely captured, and a theoretical underpinning for implementation of community participation in primary care is absent.

**Objective:**

To conduct a theoretically informed, multiperspectival empirical analysis of the implementation of community participation via primary care teams (PCTs) in Ireland.

**Methods/Design/Participants:**

Participatory learning and action (PLA) focus groups and interviews were held with 39 participants across four case study sites within a nationally funded programme designed to enable disadvantaged communities to participate in primary care. Normalization process theory (NPT) informed data generation and analysis of how diverse stakeholder groups worked together to implement community participation via PCTs.

**Results:**

The various stakeholders had a shared understanding of the value of community participation on PCTs. Motivations to get involved in this work varied, but were strong overall. Challenges to enacting community participation on PCTs included problems with the functioning of PCTs and a lack of clarity and confidence in the role of community representatives at PCT meetings. Informal appraisals were positive, but formal appraisal was limited.

**Discussion and Conclusion:**

The implementation and sustainability of community participation on PCTs in Ireland will be limited unless (i) the functioning of PCTs is strong, (ii) there is increased confidence and clarity on community representatives’ roles among all health‐care professionals, and (iii) more sophisticated methods for formal appraisal are used.

## 1 INTRODUCTION

Community participation in primary care has its origins in the Alma‐Ata Declaration of 1978, which stated that “people have the right and duty to participate individually and collectively in the planning and implementation of their health care.” It is defined as:

a process by which people are enabled to become actively and genuinely involved in defining the issues of concern to them, in making decisions about factors that affect their lives, in formulating and implementing policies, in planning, developing and delivering services and in taking action to achieve change^1^ (p. 10)

Since then, the concept of involving patients and the public in health‐care planning has gained acceptance and is enshrined in health policy across a range of international settings including the UK,^2^, ^3^ Scotland,^4^, ^5^ Wales,^6^, ^7^ Canada 8, 9 and New Zealand.^10^

There are examples internationally of individual and collective processes to implement community participation in primary care.^11^, ^12^, ^13^, ^14^, ^15^, ^16^, ^17^ In recent years, collective processes have been adopted in several countries: for example, citizens juries^14^ and patient participation groups^15^, ^16^ in the UK, citizen juries^13^ and community representatives on health service committees^18^ in Australia, dialogue sessions^19^ in Canada, mixed advisory committees (MACs)^20^ in Italy and community participation in primary health‐care organizations^21^, ^22^ in New Zealand.

In this study, we focus on collective participation in primary care, which can overcome the reductive individualistic approach to health‐care participation^20^ and create a more efficient and effective health‐care system.^23^, ^24^, ^25^, ^26^, ^27^ It has also been shown to enhance the delivery and uptake of health interventions to address health inequalities,^28^, ^29^, ^30^, ^31^, ^32^ and increase community cohesion and leadership.^31^, ^32^, ^33^, ^34^

Despite this international policy context and efforts to implement community participation in primary care, there are major gaps in our understanding of its purpose, processes and outcomes.^32^ There are limited data across the multiplicity of stakeholder perspectives on implementing community participation in primary care in practice, and community perspectives are rarely captured.^27^ Furthermore, there is a lack of evidence for how the various stakeholders work together in a primary care setting to implement community participation within interdisciplinary teams. Lack of clarity and agreement between stakeholder groups about the roles of community representatives remains a major obstacle to effective community participation.^18^, ^35^, ^36^, ^37^

Although theory has been used to understand how far patient and public involvement (PPI) was embedded within health‐care research in certain areas,^38^ there has been no use of theory to study community participation in practice despite the call for theoretically informed, empirical analysis of implementation to generate insights and transferrable lessons for community participation in primary care across settings.^39^ This is a priority for research, policy and practice.^32^

In Ireland, community participation in primary care became enshrined in health policy with the launch of the 2001 primary care strategy. This strategy sought to transfer most health‐care provision into the community to be delivered by interdisciplinary primary care teams (PCTs).^40^ PCTs were encouraged to ensure community participation in service planning and delivery. A greater input from the community and voluntary sector was proposed to enhance the advocacy role of PCTs.^40^

Despite this, and other interim measures such as the national strategy for service user involvement,^41^ involvement of patients and communities in the development and running of PCTs is not routine practice across the country,^39^, ^42^, ^43^ is hard to achieve,^44^ and is generally not regarded by service providers as an important resource for PCTs.^45^ Therefore, the aforementioned gaps in international literature are also relevant to the Irish context.^43^, ^46^

## 2 RATIONALE FOR THIS STUDY

The aim of this study was to address these international and national gaps in knowledge and to conduct a theoretically informed, multiperspectival empirical analysis of the implementation work that has taken place in Ireland to embed a programme of community participation in primary care (known as the Joint Initiative). This study focuses on the implementation of community participation on PCTs.

## 3 METHOD

### 3.1 Study context

This study took place within the Irish primary health‐care context following the end of a nationwide funded initiative—the Joint Initiative (JI)—to support community participation in primary care.

As a function of the JI, a range of community participation activities were developed including community needs assessment, health promotion and mental health awareness programmes, and community representation in the development of local primary care services^42^, ^47^ As mentioned above, the focus of this study was on collective community participation processes on PCTs in Ireland.

### 3.2 Study design

The analysis in this study is drawn from a larger qualitative retrospective case study (2011‐2014) of the JI programme. The design of the study was in accordance with Yin's recommendation for use of case studies to explore a phenomenon within its real‐life context.^48^

### 3.3 Sampling and recruitment

Following the principles of purposeful sampling,^49^ and in consultation with the external consultant who had evaluated the JI,^42^ four case study sites were chosen from the 19 JI demonstration projects to represent the geographical spread of the projects, the level of experience with community participation, the various populations involved, and the “successful” and “less successful” interactions with PCTs (see Table 1).

**Table 1:** Case study sites and research participants

Case Study (CS) Site/Joint Initiative (JI) Project description	Experience of interaction with primary care teams (PCT) at point of recruitment	Research study participants	Employment status	Data generation method used Participatory Learning and Action (PLA) focus group or interview
CS Site 1: This case study site was a migrant health forum JI project which interacted with the PCT around health issues relevant to migrants in a rural town with high deprivation and a large migrant population. The project developed a model of community participation for migrant communities based on community development principles.	The migrant group reported experiencing difficulties communicating with the PCT and did not achieve the envisaged involvement with the PCT. Participants reported that they felt failure in relation to community participation on PCTs.	Total no. of study participants N = 5 Community representatives on migrant health forum (n = 3) Project coordinator (n = 1) Health Service Executive (HSE) policy personnel: Programme Manager (n = 1)	Unpaid Paid Paid	PLA focus group Interview Interview
CS Site 2: This case study site was a JI project with a large network of people involved in community participation and primary care in a rural area with low‐density population.	This group had a long history of working in the area of community participation and had good experiences of enacting community participation on PCTs and with the larger primary care network. There was reported successful interaction with the PCT.	Total no. of study participants N = 22 Community Representatives on PCT or Community Health Forum (n = 16) HSE professionals: HSE Social Inclusion Manager (n = 1) HSE policy personnel: Programme Manager (n = 1) Primary Care Development Officer (n = 1) GPs (n = 3)	Paid (n = 6) Unpaid (n = 9) Unknown (n = 1) Paid Paid Paid Paid	PLA focus groups Interview Interview Interview Interview
CS Site 3: This case study site was a large JI inner city regeneration community health project working with disadvantaged inner city communities. This was an area with high levels of poverty, disadvantage and health inequalities. There were a large number of community groups and projects up and running in the area.	The case study site had engaged widely with groups and projects as well as with the PCT. This site had varied community participation activities across a number of health initiatives. Reported mixed success with interacting with the PCT.	Total no of study participants N = 8 Community Representatives/Community health workers (n = 4) Project Coordinator (n = 1) HSE professionals: PCT Social Worker (n = 1) Occupational Therapy (OT) Manager PCT formerly PCT Manager (n = 1) PCT OT (n = 1)	Paid Paid Paid Paid Paid	PLA Focus groups Interview Interview Interview Interview
CS Site 4: This case study site was a JI Local Development Partnership Project in a rural town with a history of working with disadvantaged communities across the community, voluntary and statutory sectors. This site had experience and expertise in community consultations and addressing rural isolation and health inequalities.	This site had reported good interaction with their PCT and with different community participation initiatives in the area.	Total no of study participants N = 4 Community Representatives/community activist (n = 1) HSE professionals: PCT Social Worker (n = 1) Development worker (n = 1) HSE policy personnel: Primary Care Development Officer (n = 1)	Unpaid Paid Paid Paid	Interview Interview Interview Interview

The research participants (n = 39) were identified and invited to participate in the study via gatekeepers at the four case study sites. Gatekeepers were paid project coordinators at each site who communicated with community representatives and health service employees and managers about the study and extended the invitation to them to participate in focus groups or interviews.

Participants were categorized as follows:

1. Community representatives^1^ who had been involved in the JI demonstration projects and had some experience of interacting with PCTs within this context (n = 27).
2. Health service executive (HSE)‐employed health‐care professionals who were working in the PCTs and worked with the JI demonstration projects (n = 5)
3. HSE‐employed service planners and policymakers who oversaw the development of PCTs and had been involved with the development and roll‐out of the JI (n = 4)
4. General practitioners (GPs) working with PCTs. (n = 3)

### 3.4 Ethical approval

The Irish College of General Practitioners (ICGP) in Ireland provided ethical approval for this study.

### 3.5 Data generation

We employed normalization process theory (NPT) to inform data generation and analysis. See Box 1.

Box 1 **Normalization Process Theory (NPT) theoretical constructs 1**

1 NPT consists of four constructs designed to explain how stakeholders understand, buy into, enact and appraise a new practice.

NPT has been applied in several areas of health services research and has proved useful to enhance understanding of implementation journeys of a variety of interventions and innovations in health‐care settings,^2^, ^3^ from a range of perspectives.^1^, ^2^, ^4^ In this case, NPT allowed us to extract and explore data pertaining to the practice of community participation on primary care teams (PCT)s across the range of stakeholders involved with the PCTs and JI projects. Data were deductively coded onto the four NPT constructs in NVivo^5^ and further analysed via the lens of levers and barriers to implementation.

1. Coherence: Can stakeholders make sense of community participation on PCTs as a new way of working? Where coherence is strong there is a shared understanding across all stakeholders of what this work will entail for individuals. There is common understanding about the value and purpose of this work.
2. Cognitive Participation: Will they engage with/“buy into” community participation on PCTs? Where cognitive participation is strong there are legitimate reasons for stakeholders to get involved and there are strong motivations for them to engage in this work. There are champions to support the work and resources available to get the work up and running.
3. Collective Action: What do stakeholders need to enact community participation on PCTs in daily practice? Where collective action is strong there is shared understanding about roles and responsibilities among stakeholders, there are resources available and structures in place to support the work in day‐to‐day practice and there are good relationships between and across stakeholders which support the work.
4. Reflexive Monitoring: Can stakeholders formally or informally appraise the impact of community participation on PCTs? Where reflexive monitoring is strong there is agreement that the work has resulted in benefits for individual and wider community, there are clear evaluation mechanisms in place and there is a shared understanding about what changes are required in structures to sustain and embed the work.

Participants were contacted via gatekeepers and chose their preferred method of data generation (ie semi‐structured interviews or participatory learning and action (PLA) focus groups). PLA focus groups and data generation methods^50^, ^51^ were used with community representative groups where possible. PLA focus groups involve the use of PLA techniques with inherent visual and analytic techniques. They were valuable because they allowed community representatives’ perspectives to be shared across and between participants and for preliminary data analysis to be conducted in a collaborative and participatory fashion.^52^, ^53^ see Box 2. These techniques have been previously used with migrants and people with aphasia.^53^, ^54^, ^55^, ^56^, ^57^

Box 2 **Participatory learning and action (PLA) techniques used for data generation and analysis**

1 The techniques used were flexible brainstorming (FBS) for data generation and Card Sorts for co‐analysis of data. The FBS is a technique used to generate as many ideas as possible related to the research question and recording them on a large chart. It is suitable for those with low literacy as there are options to use pictures from magazines, draw pictures or have the research team write or spell words for participants if needed. PLA materials included a shared blank flip chart sheet, coloured markers and coloured stickies, pens, paper, key words, symbols and pictures placed in the centre on a large table for easy access. Participants chose materials to communicate their emic experiences of enacting community participation on primary care teams. The **Card Sort** was used to begin the process of thematic co‐analysis of the data developed in the flexible brainstorm. All information placed on the chart was organized by asking “*what ideas belong together? How would you organise these so that they can be organised into meaningful ‘bundles’”?* Participants moved the material on the chart into themes all the while explaining why these ideas belonged together and cross‐checking with each other that they were satisfied with this organization of ideas.

Community representatives chose focus groups as their preferred method of data generation as these research sessions were held to coincide with their usual scheduled meetings, which was convenient and time‐efficient. Community representatives also indicated that it was a welcome means to reflect together on their community participation practices and their shared experiences of interacting with the PCT.

Interviews were favoured by health‐care professionals, GPs and HSE service planners and policymakers, allowing the participants to speak within their own conceptualization of the phenomenon of community participation in primary health care and to make this explicit.^58^

They were more convenient for this cohort of participants as the interviews were scheduled at a time and location suited to the individual and did not interrupt their busy schedules of work.

Gathering data from both focus groups and interviews provided rich narrative accounts, which were analysed for shared and differential perspectives and experiences between and among the participant groups, and across and between case study sites (Table 1).

### 3.6 Data analysis

All interviews and focus groups were recorded and fully transcribed for analysis. Participants chose a pseudonym to maintain anonymity.

Two researchers were involved in the focus groups, ET and RME. ET undertook all interviews. Data analysis for the wider project pertaining to community participation in primary health care was led by ET and deliberated in data analysis meetings with AMF and RME.

Data analysis for this study specifically focused on data pertaining to community participation on PCTs and was led by ET. Analysis was then discussed and developed with AMF and AH.

Deductive data analysis^59^ was informed by normalization process theory (NPT) using NVivo. While there were different data generation methods used, with implications for group reflection (focus groups) versus individual conceptualization (interviews), data from both methods resonated with the four constructs of NPT. This indicates that the data generation methods did not impact on the conceptual nature of the results.

**Table 2:** Levers and barriers to community participation on primary care teams (PCTs) using normalization process theory (NPT) constructs to evaluate implementation; synthesis of findings across research sites^61^, ^62^, ^63^, ^64^, ^65^

NPT construct (n = 4) (May and Finch 2009)	Lever	Barrier	Status
Construct 1: Coherence: Can stakeholders make sense of community participation on PCTs as a new way of working?	Shared views about potential value of community participation on PCTs across stakeholders directly involved in the Joint Initiative	Lack of shared understanding by wider stakeholder community about the role of community reps on PCTs	Moderate
Construct 2: Cognitive Participation: Will stakeholders engage with/“buy into” community participation on PCTs?	Champions employed by health service executive (HSE) drive this way of working forward Existing positive relationships support buy‐in Personal motivations to empower communities enhance buy‐in for community members Fits with social determinants of health or professional ethos of team members		Strong
Construct 3: Collective Action: What do stakeholders need to enact community participation on PCTs in daily practice?	Dedicated resources and funding for paid role to coordinate the work	Time‐consuming to plan and coordinate across stakeholder groups Lack of PCT readiness and PCT functioning Lack of clarity and confidence about community representatives’ roles at PCT meetings Lack of respect by some PCT members for role of the community representative	Moderate
Construct 4: Reflexive Monitoring: Can stakeholders formally or informally appraise the impact of community participation on PCTs?	Informal evaluations are broadly positive Leads to increased awareness about primary care services	Formal HSE key performance indicator (KPI) is limited and does not cover the complexity and value of the work Uncertainty about the sustainability of community participation on PCTs	Weak

Findings per construct were analysed asking “how strong is the implementation of community participation in PCTs?” There is no recognized system for this layer of NPT analysis. Therefore, a working definition for strong implementation was developed by the research team (see Box 1) and was used as a benchmark to classify the implementation as strong, medium or weak (Table 2).

Codes ascribed to participants include pseudonym, data generation method, employment status and case study site, for example *John Walsh, Interview, Paid Primary Care Development Worker, CS Site 4*.

### 3.7 Quality and rigour

Several steps were taken to increase the quality and rigour of our results.^60^ These included the following: recording of reflective notes during fieldwork, regular data analysis clinics for NPT analysis, member checking with participants via email and face‐to‐face meetings as well as feedback sessions with participants. NVivo 10 software was used to facilitate data coding and analysis and sharing data across the research team. These steps were continued until there was sufficient, thick description in the data, that is until data saturation had been reached.^60^

## 4 RESULTS

Participants (n* *=* *39) were paid and unpaid community representatives (n* *=* *27); HSE health‐care professionals working on PCTs (n* *=* *5); HSE service planners and policymakers who oversee the development of PCTs (n* *=* *4); and GPs (n* *=* *3) (see Table 1).

### 4.1 Coherence: Can stakeholders make sense of community participation on PCTs as a new way of working?

All participants in the study considered that community participation on PCTs was about meaningful reciprocal relationships between stakeholders to represent the voice of the community in primary health‐care delivery generally and at the PCT meetings more specifically.

And their [community reps’] role would be I suppose to act as a voice for the community, with regards to needs and … ultimately maybe to have some impact on shaping services. (John Walsh, Interview, Paid Development Worker, CS Site 4)

In particular, managers and policymakers saw this as a very valuable mechanism for consultation with communities. There was a general consensus that the role of community representatives was to feed ideas to the PCT about service needs of the community.

The community representatives saw the value of their role as a means to empower community members, find their voice and encourage participation. They saw their role as a catalyst for change in the dynamic of PCTs.

Just another thing that should be on there is something like empowerment. Because I think even through involvement in community forums and that people are coming into those community forums that might not have had any community participation prior to that involvement. So you know its empowering them to find their own voice within the community. (Midge, Focus group (FG), Unpaid CHF Member, CS Site 2)

However, participants in the study agreed that there is a lack of shared understanding among the wider network of PCT members and the wider community in their area about community participation on PCTs and what the role of community representatives entailed.

I'm not too sure that people [PCT members] attending the meetings really understand why they [community representatives] are there. (Mary, FG, Paid PCT Community Rep, CS Site 2)

For GPs, the introduction of community representatives on PCTs elicited a fear that they would lose control of their work, and this was a concern for them at the start of the process.

But yet when it was first mooted then that you know people were going to go out and find out what did people actually want, we thought … well are we going to lose control of our work? (Dock, Interview, GP, CS Site 2)

Overall coherence was moderate. This meant that stakeholders who were closely involved in the JI across roles and case study sites generally saw the value of community participation on PCTs, but there was not a shared understanding about what this work would entail in the wider network of stakeholders.

### 4.2 Cognitive participation: Will stakeholders engage with/”buy into” community participation on PCTs?

Buy‐in to this way of working for community representatives happened because they were invited to be a representative on the PCT by “champions of the JI” who were known to them. This was usually a community development worker, project coordinator or PCT work colleague. These “champions” were described as being passionate and committed.

But can I say Bree [paid community health forum support worker] … is very passionate in the work she does and I'm sure we'd all agree and she puts in an awful lot of work and you know only for you Bree you know she's a great, you've great management skills. You know, I think only for you, I probably wouldn't be still here. (Corrina, FG, Paid CHF Support Worker and PCT Rep, CS Site 2)

They also had personal motivations and became involved because they lived locally and had a vested interest in the area. It was also an opportunity to share information with the PCT about particular community projects with which they were involved.

I felt that I had a contribution to make, when they asked me that night why do you want to be a rep and I said I was hoping to give [something back to the community]. I had worked on a mental health group for a long time before it, and even though I knew it wouldn't be just representing in the mental health I felt I could be a voice for them [the community] as well on the team [PCT]. (Tess, FG, Unpaid PCT Community Rep, CS Site 2)

Buy‐in for health professionals was also influenced by champions of the JI and existing relationships, which supported the set‐up and roll‐out of PCTs.

So those relationships were there, and we had done an initial bit of work and I guess maybe there was reasonably high expectations of the roll‐out of primary care teams and maybe the impact it would have and maybe the opportunities for communities to become involved. (John Walsh, Interview, Paid Primary Care Development Worker, CS Site 4)

For some health professionals, it fitted with the community development model/social determinants of health and their philosophy of work in a paid professional role.

Actually primary care is a huge opportunity for social work to go back to its roots about being a community social worker, and I suppose that's one of the reasons I was particularly interested in, in primary care and in this project was that it is about those, those skills and values that social work began with, is actually engaging local communities in having a say in what they want in their own health and their own wellbeing. (Thomas, Interview, Paid Social Worker PCT, CS Site 4)

Motivation for policymakers was that community participation on PCTs connects with primary care strategy and therefore “activates” the policy on the ground.

Well, I suppose it goes back to the primary care team involvement and the national primary care strategy which obviously advocates this element (community participation on PCTs). (Paddy, Interview, Paid Primary Care Development Officer, CS Site 1)

Cognitive participation was strong. This means that stakeholders from all groups bought into this way of working because they were invited by champions, and existing relationships supported the work. There were also complementary, differential motivations for community and professional participants that fuelled interest and responsiveness to invitations to get involved in the JI.

### 4.3 Collective action: What do stakeholders need to enact community participation on PCTs in daily practice?

All participants talked about the importance of a paid role to coordinate this work.

I don't think it would happen unless somebody specifically has that role or mandate to do it because it's just understood that it will happen. (Shell, Interview, Paid Migrant Health Forum Coordinator, CS Site 1)

The training provided was also valued.

However, despite these levers, participants across sites and across stakeholder roles emphasized the barriers they experienced while trying to get this way of working into practice.

First, participants in local management roles talked about extensive planning and consultation that had to take place to maintain everyone's involvement and to organize what was going to happen, where and when:

people don't necessarily understand the amount of detail or planning or consultation that's involved in this, the HSE management or people nationally wouldn't understand that type of thing and they wouldn't understand the level of detail and the amount of time and the buy‐in and the commitment and how long it takes. (Paddy, Interview, Paid Primary Care Development Officer, CS Site 1)

Second, all participants irrespective of role and across sites felt that the PCTs were not at a stage of development for community participation to operate effectively. All participants agreed that this was a major barrier to the enactment of community participation on PCTs.

So the primary care team itself wasn't functioning, the business meeting wasn't functioning very effectively, it was very new. So there wasn't really the channel of communication about what was being expected in there and then what they can do, what they were expected to do. What happened was the primary care team continued not to function very well for a good long time, probably three years I should think after it formed. (Lydian, Interview, Paid Community Support Worker, CS Site 2)

GPs spoke about their frustration with PCT working itself, the different styles of working involved and the challenge of this for them.

looking back, I mean there was a lot of problems with it [PCT working]. Because we had meeting after meeting after meeting where we were able to make a decision here and now, if we met with the dietician or the different branches from the hospital we could make a decision about where we go here and now as GPs. But they couldn't, there was line managers, meetings about meetings about meetings. (Dock, Interview, GP, CS Site 2)

Third, GPs were also frustrated about the community development style of working, which they felt took up a lot of time and did not necessarily need their input.

Yeah, I wouldn't have the resources to travel. My own role I did it purely on a voluntary basis, I had to make up the time elsewhere. I was rushing, like there was tea and sandwiches provided which was great so I didn't have to miss my lunch, but it was a bit of a chore. (Tom, Interview, GP, CS Site 2)

Where community representatives did get to participate in PCT meetings, there was a lack of clarity among some health professionals about the precise role of the community representatives at those meetings. There were misunderstandings about issues such as loss of confidentiality at meetings, and what the community representatives were trying to achieve.

But we were trying to kind of get across the idea that the community reps weren't here to discuss specific clients, they were here to discuss broader issues and they could bring stuff to us and we could advise them of things that they could share with the community, but the team wasn't ready, that's the reality. (Thomas, Interview, Paid Social Worker PCT, CS Site 4)

Furthermore, the community representatives felt their role was tokenistic.

I suppose the only other negative impact … a negative thing would be I don't think we are seen as equal partners by the clinicians. And that is a difficulty. (Midge, FG, Unpaid CHF Member, CS Site 2)

Management felt that GPs did not appreciate the role of community representatives on the PCT.

I think the GPs particularly just were really not, they were quite happy to let us do it and maybe partake in it but they didn't see, I don't think they really saw the value or the, what this would achieve. That would be sort of my, there would be a standard approach really for my sense of it, I think they feel it's a bit fluffy and it's a bit and nothing really happens. (Carol, Interview, Paid Primary Care Development Officer, CS Site 4)

However, for GPs this “distance” was explained by their view of community participation in primary care more generally. They did not feel the need to interfere with the work of the community and just allowed community representatives to get on with it.

So a lot of the over 50s club and they had the community bus run for the elderly, so these services were run totally [by the community], we didn't really have much to do with them. We would support them and say yes it's a good idea, but the rest as a team ran with it themselves. (Dock, Interview, GP, CS Site 2)

Collective action was moderate. This means that available resources and training were important levers for enacting community participation on PCTs. However, the PCTs were not sufficiently developed for community participation to operate effectively. This impacted on relationships in the team, and community representatives did not feel that they were viewed as equal partners at the PCT meetings.

### 4.4 Reflexive monitoring: Can stakeholders formally or informally appraise the impact of community participation on PCTs?

All participants agreed that community participation on PCTs is hard to evaluate or measure. Community participation on PCTs was measured formally by a key performance indicator (KPI) (a count of the number of community representatives on the PCT) by the HSE nationally. However, this metric was considered by most participants as being very limited. It did not capture the breadth and variety of activities that comprise community participation activities. This was cited as problematic particularly among local management.

There's also the fact that there's lots of activities we are working on with primary care teams that don't form part of the official statistics … but it might not count that they went along and took part in a group activity, as part of say a health screening event at a football match. (T. Burnett, Interview, Paid PC Development Officer, CS Site 2)

When people were asked to informally appraise the impact of community participation on PCTs, the biggest benefit cited across all stakeholders and case study sites were increased awareness about services available in the community and among HSE personnel about community projects and the role of community workers.

among the primary care team it heightened the awareness of what was going on in the community. And then the flip side of that is that the community was more aware of the primary health care team and what they were about and how they functioned etc. (John Walsh, Interview, Paid Development Worker, CS Site 4)

For many community representatives on an individual level, this work led to personal benefits such as empowerment. The training and skill development that they received supported their career paths. This was particularly evident for the migrant health forum group.

On a collective level, community representatives felt that community participation on PCTs improved service delivery for the local population, resulted in more efficient use of resources and connected GPs with their community.

Yes we had huge success within the community … like the gardening and mental health programme, the green prescription, and different aspects of that, and that has been obviously through our involvement in the primary care team. That we've been able to channel some of the resources down in, you know we have that tangible success. (Tess, FG, Unpaid PCT Community Rep, CS Site 2)

They also cited mutual learning for community representatives and clinicians on the teams. They educated clinicians about the value of community participation, and this resulted in improved networking across community regions to share resources.

The impact has been educating clinicians and GPs on the value of community participation. (Bree, FG, Paid CHF Support Worker and PCT Rep, CS Site 2)

However, for some community representatives there was disappointment that nothing happened as a result of the work, and there was a sense of lost opportunity.

Ah no, I suppose there was frank discussion but I would just see that we still, at the end of the day nothing has changed. (John, Interview, Unpaid Community Activist, CS Site 4)

Similarly, GPs were generally less positive about this work and felt they had little to contribute to the community participation on PCTs process.

My difficulty was while I hope I contributed a bit, I'm not too sure how much my contribution is relevant to these community groups really. (Tom, Interview, GP, CS Site 2)

Across all participant groups, there was uncertainty about the future of community participation on PCTs. There was agreement that it is a challenge to sustain this way of working. In particular, the lack of resources to sustain the PCTs was cited as a challenge for the future. The economic recession impacted the work, and there were significant budget cuts, introduced around the time of fieldwork, which decimated the scope for continuing the work initiated by the JI and starting new projects in other settings.

I mean recently with budget cuts and restraints on people, it's just not, it's something that makes it very difficult to achieve now. In the current environment I don't know how it could be achieved because people are so stretched that this is just something else that they have to do. (Shell, Interview, Paid Migrant Health Forum Coordinator, CS Site 1)

Management in particular felt that this work needs to be built into professional roles, and there needs to be more education about the practicalities of enacting this work.

I think it's a challenge [the future of CP on PCTs] probably for both organizations now because as our resources diminish … it becomes less of a focus as other priorities take heed … I think people do value the importance of it but it just can get lost with everything else that's going on. (Carol, Interview, Paid PC Development Officer, CS Site 4)

Reflexive monitoring was weak. This means that informal appraisals of community participation on PCTs were quite positive, but it was hard to formally evaluate or measure. The scope for sustaining the work and transferring lessons learned to other sites was considered to be very poor, particularly in the context of the economic recession that decimated resources.

## 5 DISCUSSION AND CONCLUSIONS

### 5.1 Summary of key findings

There was a shared understanding about the idea and potential value of community participation on PCTs among stakeholder groups involved in the JI across roles and case study sites, but this did not hold across the wider network of stakeholders on PCTs and community.

Stakeholders across groups bought into this way of working because they were invited by passionate and convincing “champions.” Existing relationships and complementary motivations also fuelled buy‐in.

There were positive examples of enacting community participation on PCTs, supported by available resources and training. However, it was challenging because it is time‐consuming work for those in management roles. Furthermore, it was taking place against the background of poorly functioning PCTs as well as confusion and concern about community representatives’ role at PCT meetings. This thwarted health‐care professionals’ confidence in the work and inhibited meaningful engagement experiences for community representatives.

There were informal, positive appraisals of impact from most stakeholder groups. There was also consensus that impacts are difficult to capture formally and that sustaining and transferring the work that had started was going to be very difficult in the context of the economic recession.

### 5.2 Methodological critique

This study is a snapshot of a funded national initiative introduced in Ireland at a particular point in time and represents findings from four case study sites within this larger initiative. We recognize that in this study both the case and its context were changing over time. The national initiative began during an economic boom, and our fieldwork took place after a global recession that impacted considerably on Irish health care generally, and the scope for community participation in particular.

A strength of this study is that it adds the unique voice of community representatives that is absent from the literature,^27^ using methods that were valuable to elicit shared and differential views about community participation experiences. Also, by drawing on a theoretical framework for implementation, we have highlighted the levers and barriers to implementation of community participation on PCTs across the multiplicity of stakeholder perspectives not reported elsewhere. Illuminating these levers and barriers across the various stakeholder perspectives using a theoretical framework offers the opportunity for comparable analyses of similar initiatives in other health‐care jurisdictions.^66^, ^67^, ^68^

In relation to the multiperspectival analysis, the participation of more GPs in the fieldwork would have been beneficial. GPs are core members of PCTs and vital to their effective functioning. Acceptance of community representatives at PCT meetings may be dependent on their attitude. Indeed, the fact that recruitment of GPs was only possible in one case study site may tell us something about why community representatives felt they were not respected in this role by health professionals and GPs, in particular, although this would need further investigation.

### 5.3 Comparison with literature

Similar to findings about PPI in research,^38^ effective community participation on PCTs is supported by shared understanding of the moral and methodological purposes of participation, a key coordinator, a positive and engaged team based on relationships established and maintained over time and a proactive and systematic approach to evaluation. In keeping with the international literature, there was general enthusiasm for community participation in planning primary health care via PCTs across stakeholders in this Irish study.^18^ The potential benefits of community participation on PCTs, such as improved service delivery and increased awareness, were recognized.^24^, ^25^, ^26^, ^27^, ^69^

Visionary leaders who are committed to working with communities were an essential ingredient of encouraging buy‐in and commitment to community participation. Community workers acted as what have been identified elsewhere in the literature as “boundary spanners,”^70^, ^71^ which means that local people were drawn into the process and, with increased confidence, became advocates and translated and mediated between local people and professionals.^21^, ^70^

However, despite a considerable investment of resources through the JI to build capacity for this work, clarity and agreement between different stakeholder groups about the roles of community representatives was problematic, as cited elsewhere,^35^, ^36^, ^37^ and GP concerns about the potential for negative impact on their practices was reported.^21^ From an NPT perspective, this lack of clarity and confidence will undermine the workability of community participation in PCTs in practice. The challenge seems to be in reaching the full network of relevant stakeholders to enhance understanding, engagement and readiness for community participation on PCTs.

### 5.4 Implementation and enactment

It is not possible to consider community participation outside a political context.^72^ This analysis has highlighted that there were two political innovations at play In Ireland at the time of this study: the introduction of primary care teams via the primary care strategy and the introduction of community participation on PCTs via the joint initiative. The problems with full implementation of interdisciplinary team working are not unique to Ireland.^73^

From an NPT perspective, in this analysis, while this dual interplay did not seem to impact so much on *sense‐making* or *engagement* processes, it clearly impacted on the readiness of PCTs to *enact c*ommunity participation on PCTs. Put simply, community participation on PCTs, without a proper PCT structure, is hard to enact. Participants in this study were adamant that PCTs should be fully resourced and running effectively before community participation is introduced.

The implementation and sustainability of community participation in PCTs in Ireland will be limited unless the functioning of PCTs themselves is stronger, there is increased confidence and clarity on community representatives’ roles among all health‐care professionals, and more sophisticated methods for formal appraisal are employed. Future research could investigate how training in methods to enact community participation on PCTs could enable shared understanding to be achieved and clarity of roles to be developed. Evaluation strategies could be built into team processes early on to investigate impact and outcomes on PCT activities. Evaluative frameworks that capture a range of outcomes including unforeseen ones should also be developed.

## CONFLICT OF INTERESTS

None to declare.

## Note

1 Community representatives in this context were defined as: “individuals, who are ‘representing’, representative, and/or ‘consultative’ of one or more populations or affinity groups. They can be stakeholders, opinion leaders, organisers and advocates. They serve as a platform and channel for information and voices of community, communicating ideas and concepts between community and health and social services and who hold people and processes accountable” (p. 34).^41^#Department of Health and Children, *Strategy for Service User Involvement in the Irish Health Service 2008‐2013*. 2008, Health Services Executive and Department of Health and Children: Dublin.
